# Optimal use of tandem biotin and V5 tags in ChIP assays

**DOI:** 10.1186/1471-2199-10-6

**Published:** 2009-02-05

**Authors:** Katarzyna E Kolodziej, Farzin Pourfarzad, Ernie de Boer, Sanja Krpic, Frank Grosveld, John Strouboulis

**Affiliations:** 1 Department of Cell Biology, Erasmus MC, Dr Molewaterplein 50, 3015GE Rotterdam, the Netherlands; 2 Netherlands Cancer Institute, Plesmanlaan 121, 1066 CX Amsterdam, the Netherlands; 3 Institute of Molecular Oncology, Biomedical Sciences Research Center Alexander Fleming, PO Box 74145, 16602 Varkiza, Greece

## Abstract

**Background:**

Chromatin immunoprecipitation (ChIP) assays coupled to genome arrays (Chip-on-chip) or massive parallel sequencing (ChIP-seq) lead to the genome wide identification of binding sites of chromatin associated proteins. However, the highly variable quality of antibodies and the availability of epitopes in crosslinked chromatin can compromise genomic ChIP outcomes. Epitope tags have often been used as more reliable alternatives. In addition, we have employed protein in vivo biotinylation tagging as a very high affinity alternative to antibodies. In this paper we describe the optimization of biotinylation tagging for ChIP and its coupling to a known epitope tag in providing a reliable and efficient alternative to antibodies.

**Results:**

Using the biotin tagged erythroid transcription factor GATA-1 as example, we describe several optimization steps for the application of the high affinity biotin streptavidin system in ChIP. We find that the omission of SDS during sonication, the use of fish skin gelatin as blocking agent and choice of streptavidin beads can lead to significantly improved ChIP enrichments and lower background compared to antibodies. We also show that the V5 epitope tag performs equally well under the conditions worked out for streptavidin ChIP and that it may suffer less from the effects of formaldehyde crosslinking.

**Conclusion:**

The combined use of the very high affinity biotin tag with the less sensitive to crosslinking V5 tag provides for a flexible ChIP platform with potential implications in ChIP sequencing outcomes.

## Background

Affinity tags have been widely used for the study of protein interactions and the isolation of protein complexes. Such tags are also increasingly used in ChIP assays in detecting the in vivo binding of transcription factors and associated co-factors to their target genes in chromatin. In searching for the optimal affinity tag for ChIP applications, three criteria are important: (a) tags must have high binding affinity; (b) tags should be preferably small and not strongly charged so as to minimize possible interference with transcription factor function (c) tags should be fairly insensitive to formaldehyde fixation. The latter is true for most tags that contain no or few lysine, arginine or histidine residues [1-3].

The biotin/(strept)avidin affinity system fulfils the above criteria due to its unique characteristics [4], which include: (a) the very tight and specific binding of biotin by avidin (or streptavidin) which, with a K_d _of 10^15 ^L*mol ^-1^, is one of the highest non covalent interactions known in nature, close to almost 10^3 ^– 10^6^times greater than the interaction of epitopes with their specific antibodies. Once formed, the biotin-streptavidin complex is not disturbed by changes in pH, introduction of detergents or high salt concentration, thus remaining stable even under very stringent washing conditions; (b) biotin is a very small molecule and is not known to affect the biological activity of tagged proteins [5,6]; (c) there are few (mostly cytoplasmic) naturally biotinylated proteins in mammalian cells, as a result the non-specific background binding of nuclear extract is low [7].

We have previously used [7,8] a short (23aa) biotinylatable tag [9,10] for the purification of GATA-1 protein complexes from nuclear extracts of erythroid cells. GATA-1 is a DNA sequence-specific zinc finger transcription factor that is essential for the differentiation of erythroid, megakaryocytic, eosinophil and mast cell lineages [11,12]. N-terminally tagged GATA-1 was co-expressed with the E. coli BirA biotin ligase in mouse erythroleukemic (MEL) cells and subsequently purified from nuclear extracts together with interacting proteins by high affinity binding to streptavidin beads [7]. In this way, a number of known and novel GATA-1 protein partners were identified [8]. We also tested the utility of the biotin tag and streptavidin binding in ChIP assays and provided preliminary evidence that it can be successfully applied in place of antibodies in ChIPs of GATA-1 target genes [7,13]. Subsequent work in other labs has provided further supporting evidence for the application of biotinylation tagging in ChIP and Chip-on-chip assays [14-16]. Thus, despite the fact that biotin contains groups that are crosslinkable by formaldehyde, it can be successfully employed in ChIP assays

In this manuscript we present steps for improving the efficiency of biotinylation tagging in ChIP applications, using biotin-tagged GATA-1 in combination with known target genes [8] as an example. We first show that different streptavidin beads are not equally efficient in ChIP assays. We also show that effective blocking with fish skin gelatin and omission of SDS during chromatin sonication are important factors in reducing background signals, which is a major concern in ChIP using complex chromatin from mammalian cells. Furthermore, we explored the utility of double affinity tags in ChIP assays. Different tags may be used in tandem, separated by a protease cleavage site to allow for differential purification using either tag or for two sequential affinity purification steps using both tags to lower the background of non-specific proteins. At the same time, this approach can greatly enhance the ability to purify the complexes to homogeneity (by using several factors of the same complex differentially tagged and co-expressed) for other applications. To these ends, we combined the biotin tag with the V5 tag and show that the V5 tag antibody mediated ChIP is as efficient as the biotin streptavidin ChIP. These results have important implications when it comes to selecting an optimal strategy for genomic ChIP and proteomic analyses of transcription factor functions.

## Results

**Figure 1:**
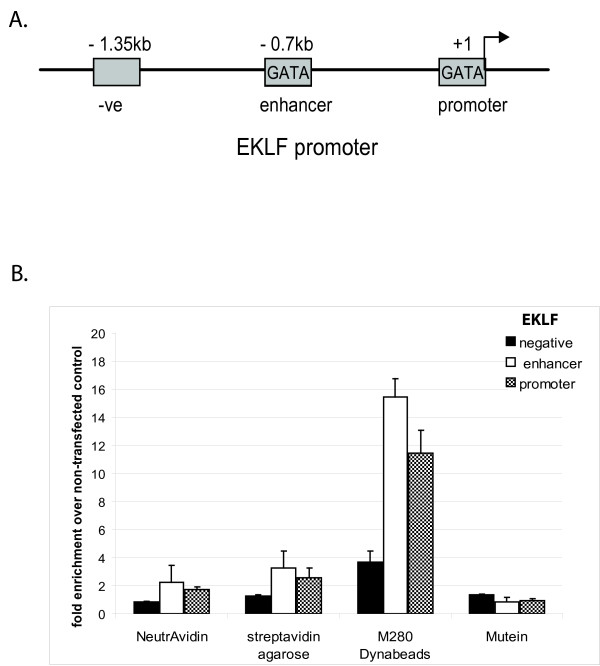
**A) Location of the ChIP primers in the EKLF gene**. "GATA" boxes indicate GATA-1 binding sites. B) Comparison of different derivatives of immobilized streptavidin: NeutrAvidin, streptavidin agarose, streptavidin mutein and M280 Dynabeads. Relative enrichment for EKLF sequences was calculated over negative control chromatin isolated from cells expressing BirA biotin ligase, but not tagged GATA-1.

We previously showed using biotin-tagged GATA-1 that streptavidin binding of crosslinked chromatin can substitute for antibodies in enriching for GATA-1 target genes in ChIP assays [7]. Due to the potential advantages offered by the very high affinity of streptavidin for binding to biotin and the importance of having multiple tags that can be used in the same cell, we wanted to extend these observations in developing an optimized protocol for the streptavidin binding of chromatin from cells expressing biotin-tagged GATA-1. In doing so, we used the EKLF and c-myb promoters as examples of GATA-1 gene targets that are upregulated or repressed, respectively, in erythroid cells. Figure 1A (EKLF) and Additional File 1A (myb) show the location of primers used for the EKLF and myb promoters and negative control sequences. Primer sequences are listed in Table 1.

**Table 1:** 

**Primer name**	**Sequence**
Myb prom FOR	ACTGCAGGGGCGCCAGATTT

Myb prom REV	GGAGAAAGGGGAGGAGAAGGAGGTA

Neg myb FOR	GAAGTAGAGGCAGGATAATCAGGAA

Neg myb REV	AGGATGAACCAGGGCTAATGC

EKLF Upstream FOR	CTGGCCCCCCTACCTGAT

EKLF Upstream REV	GGCTCCCTTTCAGGCATTATC

EKLF Promoter FOR	TATCGCACACACCCCTCCTT

EKLF Promoter REV	CCCACATCTGATTGGCTGTCT

Neg EKLF FOR	TGCTCCCCACTATGATAATGGA

Neg EKLF REV	GCCACAACCAAAGAAGACATTTT

Necdin FOR	GGTCCTGCTCTGATCCGAAG

Necdin REV	GGGTCGCTCAGGTCCTTACTT

Amylase FOR	CTCCTTGTACGGGTTGGT

Amylase REV	AATGATGTGCACAGCTGAA

### Comparison of different types of streptavidin beads

Biotinylation of biological substrates is frequently used in a variety of different applications and hence many manufacturers offer a wide range of immobilized streptavidin matrices. We have previously used paramagnetic M280 streptavidin Dynabeads for the isolation of protein complexes [7,8,17-19]. We also tested the performance of M280 beads in chromatin immunoprecipitations and compared them to three other available matrices: streptavidin agarose, streptavidin mutein and NeutrAvidin. Streptavidin agarose was used to test whether an immobilization matrix different to that of paramagnetic particles would give better yields. NeutrAvidin is a streptavidin derivative without carbohydrate side chains which is predicted to reduce background binding. Streptavidin mutein is a mutated recombinant streptavidin which binds biotin with a lower affinity thus allowing elution of bound material under gentler conditions by using biotin. Chromatin from biotin-tagged GATA-1 cells and control cells expressing BirA ligase only, was bound to different types of beads under identical conditions (overnight binding and subsequent washes). Biotin-tagged GATA-1 was eluted from the beads by decrosslinking, except for the mutein beads where we used biotin for elution. We find that the M280 streptavidin Dynabeads are the most efficient in capturing biotin-tagged GATA-1 bound to the EKLF (Figure 1B) and myb (Additional File 1B) promoters. Using M280 beads, we also find clear enrichment of GATA-1 binding to regulatory elements of the repressed GATA-2 locus (Additional File 2B). As a result, the M280 Dynabeads were used in all subsequent experiments.

### Pre-clearing chromatin

**Figure 2:**
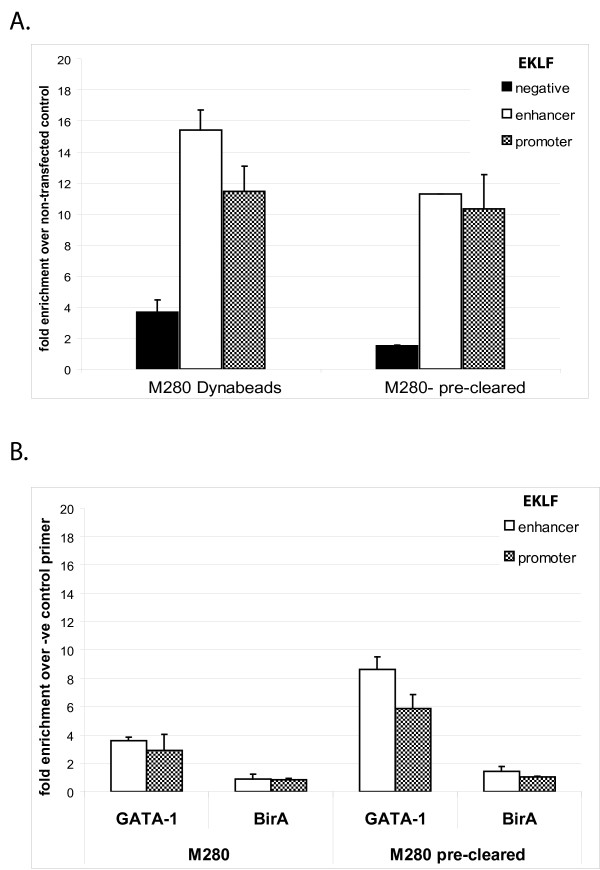
**Preclearing chromatin with Protein G Dynabeads**. A) Relative enrichment of EKLF sequences calculated over chromatin from control cells. The enrichment of the specific EKLF promoter elements appears lower after preclearing presumably due to some loss of chromatin in the additional preclearing step. B) Relative enrichment of biotin-tagged GATA-1 binding at EKLF promoter and enhancer calculated over the negative control sequence for biotin-tagged GATA-1 chromatin and chromatin from BirA expressing cells as negative control.

Pre-clearing of chromatin is one of the methods used to decrease background binding in ChIP assays using antibodies. We tested this by preclearing chromatin with Protein G paramagnetic beads (Dynal) for 1 hour at 4°C. As shown in Figure 2, this resulted in lower background and improved enrichment of EKLF sequences bound by biotin tagged GATA-1. Similar results were also obtained with the c-myb promoter (Additional File 1C).

### Blocking with fish skin gelatin

**Figure 3:**
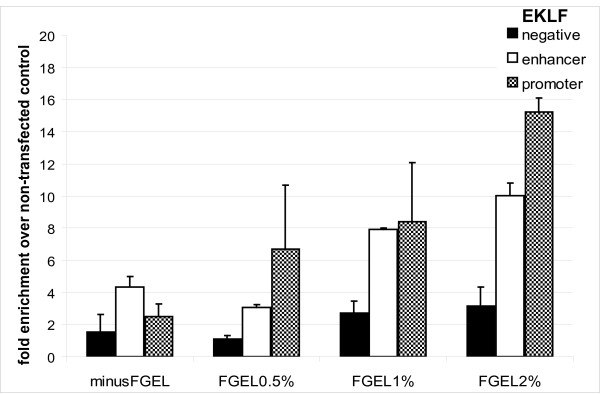
**The effect of using different concentrations of FGEL in blocking the beads**. Relative enrichment was calculated for EKLF sequences over chromatin from non transfected control cells. Low enrichment of the EKLF promoter and enhancer sequences is observed when blocking with salmon sperm DNA alone (minus FGEL control) due to the high background binding obtained with chromatin from the non-transfected control cells.

Among various blocking compounds (e.g. BSA, Chicken Egg Albumin etc.) fish skin gelatin (FGEL) has been shown to be very effective for blocking in Western and ELISA experiments [20,21]. In addition, FGEL is a very inexpensive reagent compared to other blocking reagents such as BSA. We therefore investigated whether the use of FGEL for blocking would improve the performance of M280 beads in a streptavidin ChIP. Figure 3 shows that addition of as little as 0.5% (final concentration) of FGEL (together with salmon sperm DNA) can significantly improve the yield of EKLF target sequences bound by GATA-1. Similar results were also observed with the c-myb promoter and GATA-2 locus sequences (Additional Files 1C and 2, respectively). Thus, blocking the beads for 1 hour with FGEL and salmon sperm DNA reduces the background compared to blocking with salmon sperm DNA alone. In addition to blocking the beads, we also added 1% FGEL to chromatin during binding to the beads and obtained similar results to those when FGEL was used for blocking the beads only (not shown). As a result, we have included 1% FGEL in blocking the beads in all subsequent experiments.

It has been shown previously that biotin tagging allows more stringent washes (containing up to 3% SDS) compared to other affinity tags [15]. For example, urea and thiourea are reagents widely used in proteomics to resuspend hydrophobic proteins. We therefore tested whether the background binding of hydrophobic proteins can be reduced by washing in urea/thiourea/SDS. We found that the additional wash did not significantly lower the background or increase the specific binding signals of the EKLF or c-myb promoters (data not shown) and this parameter was not investigated further.

The non-covalent binding of biotin to streptavidin is one of the strongest known in nature [22,23]. However this presents a drawback when eluting bound chromatin from the beads as is usually done in ChIP using antibodies. We were indeed unsuccessful in eluting the biotinylated protein from the streptavidin beads. The only way it could be removed was by boiling, which may result in some background due to the co-elution of non-specifically bound proteins. Alternatives would be the inclusion of protease (TEV or PreScission) cleavage sites [24-27], or the use of double tags (see below).

### Sonication without SDS

Most ChIP protocols, including those used in our laboratory, are based on the Upstate (now Millipore) ChIP protocol which includes sonicating chromatin in a buffer containing 1% SDS. Addition of SDS introduces stringent conditions and helps prevent the aggregation of insoluble protein complexes. However, high SDS concentration may affect optimal binding of chromatin by the antibody or beads and, in some approaches, inclusion of SDS is not compatible with further experimental procedures, for example in chromatin fractionation by CsCl gradient centrifugation [28,29].

**Figure 4:**
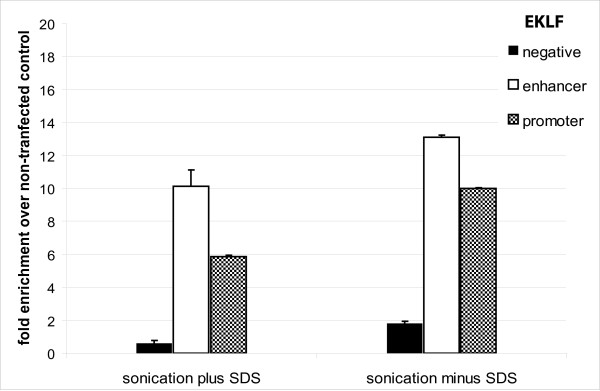
**Comparison of sonication buffer with or without the addition of SDS**. Relative enrichment of EKLF sequences was calculated over chromatin from control cells.

**Figure 5:**
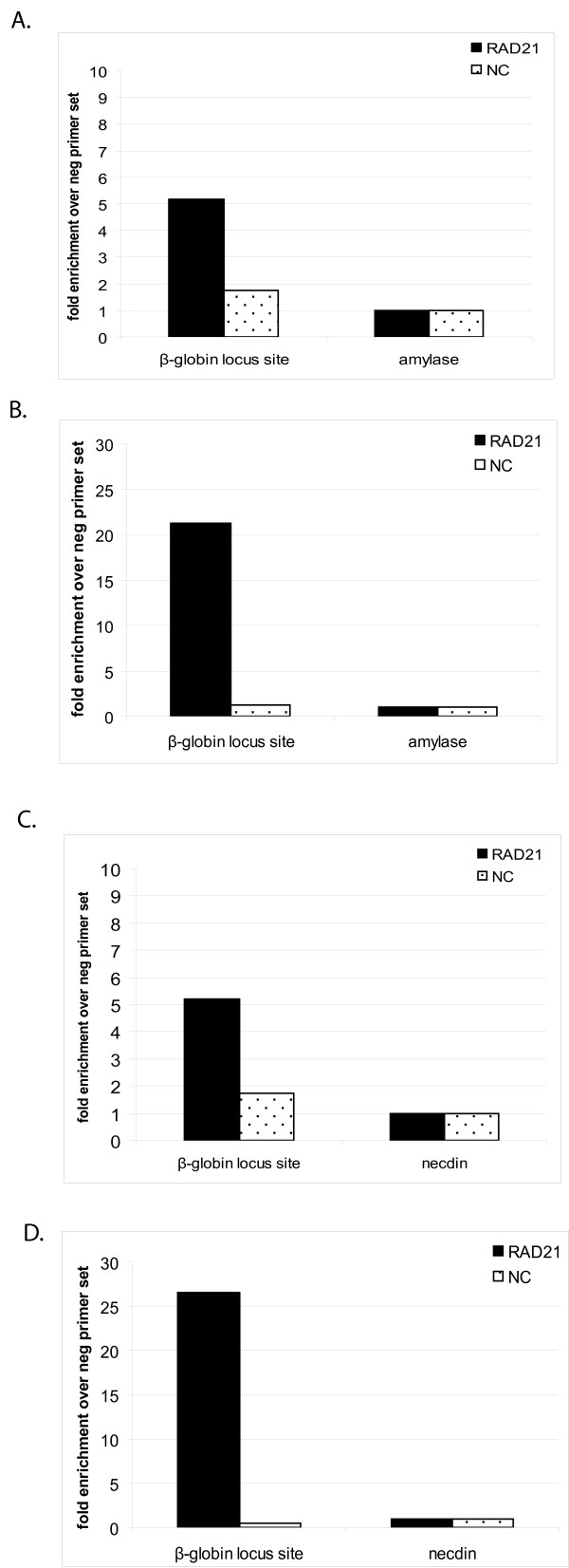
**A) Chromatin from I/11 cells prepared with SDS in sonication buffer**. Binding of RAD 21 and negative control (NC) to a β-globin site and the amylase gene (control for non-specific binding). B) Chromatin from I/11 cells prepared without SDS in sonication buffer. Binding of RAD 21 to specific site in β-globin locus is significantly improved in comparison to the result in panel A. C) Chromatin from I/11 cells prepared with SDS in sonication buffer. Binding of RAD 21 and negative control (NC) to a β-globin site and the necdin gene (control for non-specific binding). D) Chromatin from I/11 cells prepared without SDS in sonication buffer. Binding of RAD 21 to specific site in β-globin locus is significantly improved in comparison to result in panel C. Note that the binding of negative control (NC) to β-globin locus site is reduced.

We therefore tested whether sonicating chromatin without SDS would improve the efficiency of a streptavidin ChIP. Omission of SDS did not affect the efficiency of DNA shearing. Sonicating chromatin without SDS resulted in higher enrichment of the EKLF (Figure 4) and c-myb (Additional File 1D) promoter sequences, albeit with a small increase in the background binding of the EKLF negative control sequence. Thus, omitting SDS from the sonication buffer improves the yield of a streptavidin pull-down significantly. We next tested whether the omission of SDS would also improve a ChIP in a regular antibody precipitation. For this purpose we tested the precipitation of a RAD21 gene target which, under the standard SDS conditions, can be enriched 4–5 fold over background. This represents a borderline enrichment for further analysis by ChIP-sequencing (SK unpublished). When a RAD21 ChIP to a site in the β globin locus in I/11 erythroid cells was carried out with or without SDS, the sample without SDS gave a considerable improvement in enrichment (Figure 5).

### Comparison of biotin and V5 epitope tags to anti GATA-1 N6 and M20 antibodies

**Figure 6:**
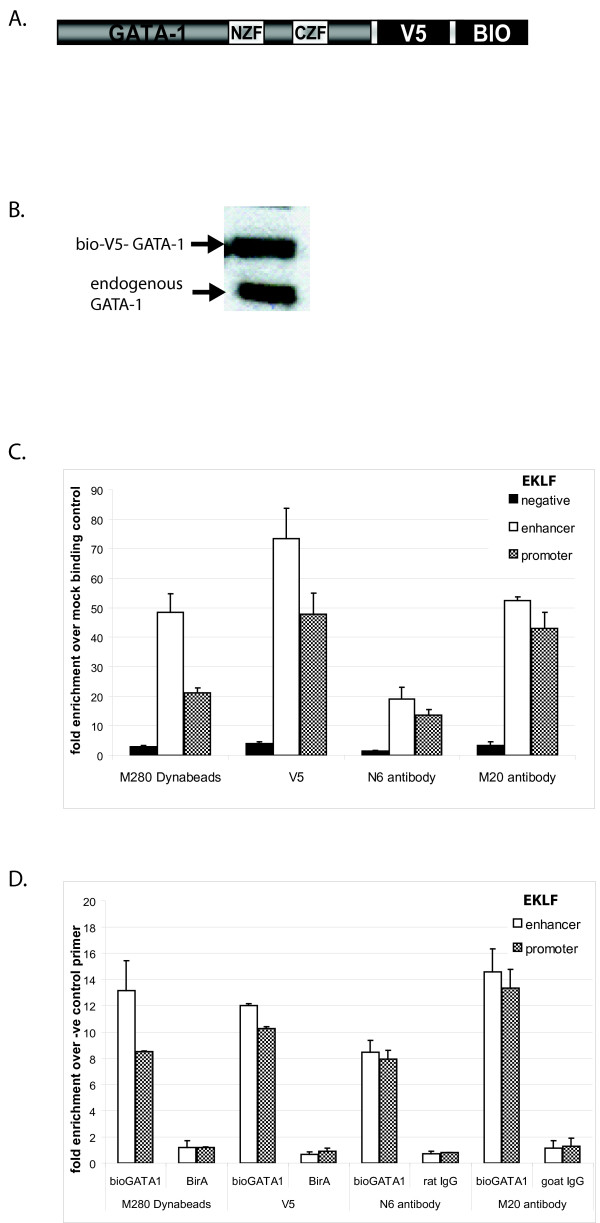
**A) Schematic representation of the C-terminally biotin-V5-tagged GATA-1**. NZF and CZF: N-terminal and C-terminal zinc fingers, respectively. V5 and biotin (BIO) tags are not drawn to scale. B) Western blot detected with anti-GATA-1 N6 antibody showing the relative amounts of biotin-V5-GATA-1 and endogenous GATA-1 in the cells used for ChIP. C) Comparison of V5, M280 and two different anti-GATA-1 antibodies, N6 and M20, tested for the binding of GATA-1 to the EKLF promoter. The enrichment is calculated over a BirA-only transfected control or over IgG negative control, respectively. V5 ChIP gives the highest yield in the EKLF enhancer and promoter elements, streptavidin ChIP with M280 Dynabeads gives comparable yield in the upstream enhancer element as M20 anti-GATA-1 antibody. The M20 antibody can enrich for more GATA-1 bound to the EKLF promoter than the M280 Dynabeads. The N6 antibody precipitates the least amount of GATA-1 bound to EKLF promoter elements. D) Comparison of V5, M280 and two different anti-GATA-1 antibodies N6 and M20, tested for the binding of GATA-1 to the EKLF promoter. Enrichment of the specific binding to EKLF promoter and enhancer was calculated over the negative primer set (-1.35 kb element in EKLF promoter). V5 agarose and M280 Dynabeads bring down comparable amounts of GATA-1 bound to EKLF enhancer and promoter sequences. The M20 antibody enriches the most for GATA-1 bound to the EKLF upstream enhancer, though this also included sequences in vivo bound by endogenous GATA-1 protein which can not be bound by M280 or anti-V5 beads. Rat and goat IgGs as well as BirA control show similarly low enrichments of specific primer sets.

The experiments described above were carried out with an N-terminally biotin-tagged GATA-1 [7]. We also generated a second construct containing a tandem affinity tag created by fusing the short (14 aa) biotin tag [10] with the 14aa V5 tag to the C-terminus of GATA-1 (Figure 6A). V5 is a short peptide sequence derived from the C-terminus of the P and V proteins of Simian virus 5 [30,31]. This construct can be used in the two-step affinity purification of tagged protein complexes, thus reducing background binding. Alternatively, one of the tags can be used on its own in cases where the second tag is inefficient, for example due to reduced accessibility in crosslinked chromatin. These are important considerations in ChIP experiments particularly in applications involving ChIP-on-chip or ChIP sequencing. This construct also allows comparison with the streptavidin-ChIP results obtained with the biotin tag fused to the N-terminus of GATA-1.

We obtained a MEL stable transfectant expressing GATA-1-V5-bio at approximately equal levels to the endogenous GATA-1 protein (Figure 6B). We compared the efficiency of ChIP by streptavidin binding or V5 antibody immunoprecipitation to two anti-GATA-1 antibodies: the N6 rat monoclonal antibody against the N-terminus of GATA-1 and the M20 goat polyclonal antibody against the C-terminus of GATA-1. We find that both the streptavidin pull-down with M280 Dynabeads and the ChIP with V5 antibodies are at least as good as or more efficient in enriching for EKLF sequences compared to the anti-GATA-1 N6 and M20 antibodies, when compared to IgG controls or chromatin from cells expressing BirA only (Figure 6C and 6D).

The V5-ChIP also works very efficiently, as it gives 12-fold higher enrichment in specific binding to the EKLF enhancer in comparison to the non-specific binding to non-related sequence (Figure 6C). In fact, the V5 tag appears to work at least as good as streptavidin binding in ChIP. When normalising to control cells expressing only BirA, the V5 ChIP gives actually a slightly better enrichment compared to the streptavidin ChIP (Figure 6C), albeit with a slightly higher background binding to the negative control sequence (Figure 6C and 6D). However, we cannot exclude that the elution from the anti-V5 agarose beads is more efficient than that from the M280 streptavidin beads.

### Formaldehyde crosslinking affects the biotin-tag more than the V5 tag

**Figure 7:**
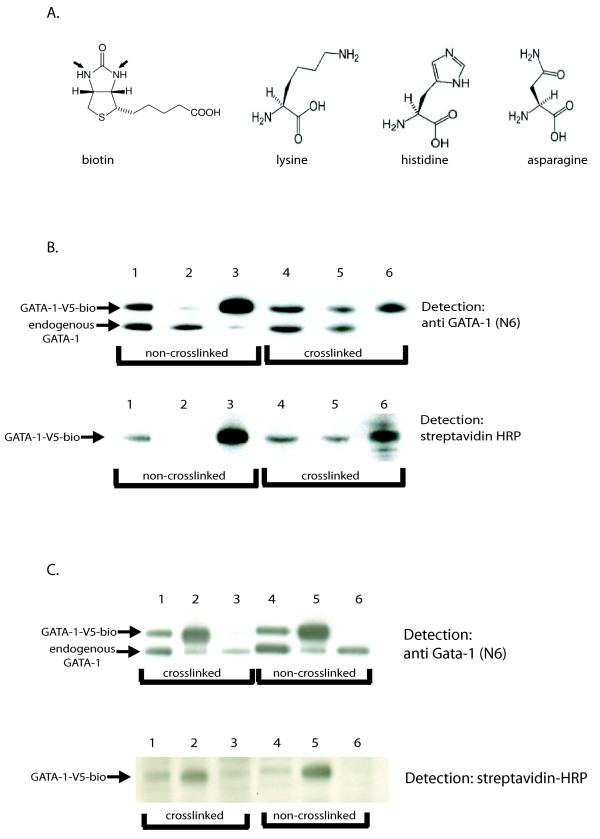
**A) Structure of the formaldehyde sensitive amino acids and sensitive groups in biotin (arrows)**. B) Western blot analysis of two binding experiments where non-crosslinked nuclear extracts (lanes 1, 2, 3) and formaldehyde crosslinked chromatin (lanes 4, 5, 6) were tested. Input and supernatant fractions (lanes 1 and 2, 4 and 5 respectively) represent 1% of total material, while bound lanes (3 and 6) represent 25% of total material. The biotin-V5-tagged GATA-1 was detected with N6 anti-GATA-1 antibody (top panel). After stripping, the same membrane was incubated with streptavidin-HRP (lower panel). C) Western blot showing comparison of binding of two different extracts: crosslinked chromatin (lanes 1–3) and non-crosslinked nuclear extract (lanes 4–6) precipitated by anti-V5 agarose. The biotin-V5-tagged GATA-1 was detected with N6 anti-GATA-1 antibody (top panel), stripped and re-probed with streptavidin-HRP (lower panel). Input and supernatant fractions (lanes 1 and 3, 4 and 6 respectively) represent 1% of total material, while bound lanes (2 and 4) represent 50% of total material. Note that V5-tagged GATA-1 is completely depleted in both experiments as the supernatant lanes are empty.

Formaldehyde cross-linking as first introduced by Salomon et al in *Drosophila *[32], has been widely used to study the binding of proteins to DNA elements in intact cells. Formaldehyde crosslinks proteins primarily through lysine, glutamine, asparagine, arginine, tryptophan, tyrosine and histidine residues [1]. Biotinylation tagging takes place through the addition of a biotin moiety to a single lysine residue present in the peptide tag, thus rendering this lysine residue unavailable for crosslinking, However, the biotin molecule has two nitrogens in the ring structure that are crosslinkable (Figure 7A). With this in mind, we compared how the biotin tag or the V5 tag, a 14aa long tag containing lysine and asparagine residues (GKPIPNPLLGLDST), are affected by formaldehyde crosslinking. To this end, the extracts containing equal amounts of GATA-1-V5-bio (see input lanes 1 and 4 in figure 7B, top panel) were bound under identical conditions. Input, bound and unbound fractions were loaded on an SDS-PAGE gel and the western blots were detected with an anti GATA-1 (N6) antibody followed by detection with streptavidin-HRP. The results showed that the binding of crosslinked material to the M280 Dynabeads was good but less efficient compared to non-crosslinked protein extract, since there was more GATA-V5-bio found in unbound fraction (compare the amounts of GATA-1 in lane 2 and lane 5 of unbound fractions 7B, top panel). In addition, anti-GATA-1 antibody as well as streptavidin-HRP detection (Fig. 7B, both panels) showed that there was less GATA-1-V5-bio bound to the M280 Dynabeads in cross-linked extract pull-down than in non-crosslinked material (compare lanes 3 with 6 in Figure 7B, both panels). These results would be expected considering that some of the biotin tagged GATA-1 protein would be inaccessible for binding by M280-streptavidin beads in the crosslinked chromatin, as has also been observed with epitope access by antibodies in ChIP.

We next tested how formaldehyde crosslinking affects binding to anti-V5 agarose. The western blot data presented in Figure 7C suggest that crosslinked and non-crosslinked material can be efficiently bound by anti-V5 beads (Figure 7C top panel, compare lanes 2 with 3 and 5 with 6). The data suggest that the efficiency of binding of crosslinked chromatin using the V5 tag is higher than that of streptavidin ChIP (compare lanes 2, 3 of Figure 7C top panel with lanes 5, 6 of Figure 7B top panel). However, efficient recovery of chromatin from the V5 beads relies on efficient elution with the V5 peptide. In addition, in other cases we have not seen this difference (E. Soler, C. Andrieu, unpublished observations) suggesting that the structure of the target protein may also have an influence.

## Discussion

We describe optimised conditions for the application of streptavidin-ChIP using crosslinked chromatin from cells expressing a biotin-tagged transcription factor. We show that pre-clearing chromatin and blocking streptavidin beads with fish skin gelatin reduces the background binding. In addition, avoiding SDS in the sonication buffer appears to increase enrichment for bio-GATA-1 binding to specific DNA sequences. We also compared streptavidin-ChIP to antibody ChIP using two different anti-GATA-1 antibodies and showed the former to be more efficient. The real difference in efficiencies between streptavidin-ChIP and antibody ChIP is even bigger because the anti-GATA-1 antibodies precipitate both the tagged and endogenous GATA-1, whereas streptavidin binds only tagged GATA-1. This means that the enrichments obtained with antibodies should be hypothetically approximately twice as high as with affinity tags pull-downs. The same applies for the efficiency of V5 ChIP, since, as mentioned above, the relative levels of bio-V5- tagged GATA-1 and endogenous GATA-1 in the extract used in this experiment are similar (Figure 6B). Thus, the results obtained with anti-GATA-1 antibodies are an overestimate when compared to the tag-based ChIPs described here.

A potential drawback of biotinylation tagging in streptavidin ChIP is related to previous reports of histones being naturally biotinylated [33]. Though it remains unknown what proportion of histones is biotinylated in vivo, the fact that previous studies have shown nuclear biotin to account for less than 1% of total cellular biotin [33,34] suggests that it is very little. In addition, we have performed extensive protein analyses by mass spectrometry and, whereas we find histones co-purifying with biotin tagged transcription factors, they do not represent any significant proportion of the background, nor did we notice an increase of histone peptides upon expression of BirA in cells [7]. Similarly, the purification of a histone variant protein complex by biotinylation tagging and streptavidin purification showed negligible histone binding [35]. Lastly, biotinylation tagging has been employed in ChIP-on-chip approaches of transcription factors and of the histone H3.3 variant, again with no evidence of background due to endogenous histone biotinylation [14,36]. Taken together, this evidence suggests that any background due to histone biotinylation is likely to be very low. In fact, one of the advantages of employing biotinylation tagging of different factors with streptavidin ChIP is that background will be the same in all cases, whereas different antibodies present with different backgrounds.

We also provide evidence that a tandem affinity tag composed of the biotin tag and the V5 epitope tag works very efficiently in ChIP. The biotin tag binding is fast and very tight, while the binding to V5 epitope is reversible (bound material can be eluted from the beads using V5 peptide), thus each tag can be used to advantage. On the basis of this evidence, we propose a scheme whereby optimal binding of chromatin, under the conditions described here, first by anti-V5 agarose, followed by elution using V5 peptide and re-binding by streptavidin beads, followed by elution/reversal of crosslinks provides a convenient and rapid purification method. Our preliminary ChIP-sequencing data (E. de Boer, unpublished) show that biotin and V5 tagging can both be very effectively used for transcription factor target sequence mapping and that the (very low) background seen in these experiments contains mostly non-specific DNA fragments that can be easily distinguished from specific target sites.

## Conclusion

The optimal conditions for streptavidin ChIP described here and the use of biotin-V5 tandem affinity tagging of transcription factors offers an easy, rapid and effective way for comparative and functional studies of different transcription complexes.

## Methods

### Cells and constructs

MEL cells were cultured as previously described [35]. Constructs and stably transfected cell lines were described previously [7]. I/11 cells were cultured as previously described [37].

### Chromatin crosslinking

Approximately 2 × 10^7 ^induced MEL cells were crosslinked with 1% formaldehyde for 10 minutes at room temperature and processed for sonication essentially as described in the Upstate (now Millipore) protocol . Chromatin was sonicated on ice with a Sanyo Soniprep 150 sonicator at amplitude 6 using 10 cycles of 15 sec "on" and 45 sec "off" to a DNA fragment size in the range of 300 to 800 nucleotides. The alternative "no SDS" sonication buffer is: 10 mM Tris, 1 mM EDTA and 0.5 mM EGTA. All buffers were supplemented with Complete Protease Inhibitor Cocktail (Roche). Aliquots of sonicated chromatin of 10 × 10^6 ^cells were stored at -80°C.

### Streptavidin ChIP

Chromatin pull-downs with streptavidin beads were carried out overnight using 20 μl of streptavidin Dynabeads M280 (Invitrogen) or 20 μl of UltraLink Immobilized NeutrAvidin Protein (Pierce) per chromatin aliquot. For streptavidin agarose (Sigma) or Streptavidin mutein (Roche) chromatin pull-downs, 60 μl of agarose slurry or beads were used per aliquot. All the beads/slurry were blocked with 400 μg sonicated salmon sperm DNA for 1 h at 4°C. Pre-clearing of chromatin prior to binding to M280 streptavidin beads was done using 20 μl of Protein G Dynabeads (Invitrogen) preblocked with salmon sperm DNA. Chromatin incubation with beads was carried out in a total reaction volume of 1 mL supplemented with Complete Protease Inhibitor, at 4°C overnight on a rotating wheel. After binding, beads were washed with 1 mL of low salt, high salt, LiCl and TE (10 mM Tris-HCl pH 8.0, 1 mM ETDA) wash buffers, 3–5 minutes each. An additional urea wash (5 M urea/2 M thiourea/1% TritonX100) was carried out after LiCl buffer wash and before the TE washes (see Results). After the washes, bound chromatin was eluted by resuspending Neutravidin, Mutein, streptavidin agarose and M280 beads in 500 μl 0.1 M sodium carbonate, 1%SDS, 0.2 M NaCl elution buffer transferring to a fresh tube and decrosslinking with shaking at 65°C for at least 5 h. Thermal elution of chromatin from M280 Dynabeads was carried out by resuspending beads in 500 μl 95% formamide, 0.1 M sodium bicarbonate and boiling at 95°C for 10 min. M280 Dynabeads were subsequently separated from the buffer using a magnetic rack. Eluted chromatin was transferred to a fresh tube and decrosslinking was carried out as described above. Decrosslinked samples were deproteinized as described by the Upstate protocol. DNA was recovered by phenol/chloroform/isoamyl alcohol extraction and isopropanol precipitation using 20 μg glycogen as carrier.

### Antibody ChIP

GATA-1 ChIP has been previously described [8]. Anti-GATA-1 antibodies used were N6 and M20 (Santa Cruz), anti-RAD21 antibodies were ab992-50 (Abcam). Anti GATA-1 antibody immunoprecipitates were eluted from the beads by incubation in elution buffer (0.1 M sodium carbonate, 1%SDS) twice for 15 minutes each at room temperature. The V5 ChIP was carried out with V5 beads (Sigma A7345). 60 μl of beads were spun and taken up in 1 ml PBS containing 200 μg sonicated salmon sperm DNA and 1.5% fish skin gelatin (Sigma G7765) mixed for 1 hour RT and washed with PBS. Diluted chromatin was taken up to 1 ml with Upstate ChIP dilution buffer and bound overnight to the blocked V5 beads at 4°C on a rotating wheel. Washing was carried out as described in the Upstate protocol . Elution was done in 40 μl HENG(10 mM HEPES-KOH pH 9.0, 1.5 mM MgCl_2_, 0.25 mM EDTA, 20% glycerol), 250 mM KCl, 0.3% NP40 and 0.5 mg/ml V5 peptide repeated 3 times 20 min each. Eluates were pooled.

### Blocking with Cold Sea Fish Skin Gelatin

A 45% Fish Skin gelatin (FGEL) stock solution (Sigma G7765) was used to block beads at 0.5%, 1% or 2% final concentrations together with sonicated salmon sperm DNA by incubation for 1 h at room temperature. Where indicated, FGEL was also added to chromatin and beads to a 1% final concentration during overnight binding.

### Real time PCR

This was done in an Opticon I (MJ Research) thermal cycler using SYBR Green and Platinum Taq polymerase (Invitrogen) as described previously [8]. Primers (listed in Table 1) were designed using Primer Express 2.0 (PE Applied Biosystems). For each experiment at least two runs were done with each sample loaded in duplicate. PCR conditions: 95°C for 10 min, 40 cycles of 30 sec at 95°C, 60 sec at 60°C, 15 sec at 75°C. Enrichment for a specific DNA sequence was calculated using the comparative C_T _method, as previously described [38]. The enrichment of bound DNA over input is calculated using the formula 2^Ct(IP)-Ct(Ref)^. Enrichment over the negative primer set or negative control chromatin from MEL cells expressing BirA only was subsequently calculated by dividing.

### Nuclear extracts and immunoblotting

These were done as previously described [7].

## Authors' contributions

KK carried out the streptavidin ChIP optimization experiments and drafted the manuscript. FP and EdB did the experiments involving the tandem biotin and V5 tags. SK did the Rad21 ChIP. FG and JS conceived of the study, participated in its design and coordination and co-authored the manuscript. All authors read and approved the final manuscript.

## Supplementary Material

Additional file 1 **GATA-1 ChIP of the myb promoter.** A) Location of ChIP primers in the myb promoter. B) Comparison of different derivatives of immobilized streptavidin: NeutrAvidin, streptavidin agarose, streptavidin mutein and M280 Dynabeads. Relative enrichment is calculated over non-transfected BirA control cells. C) The effects of preclearing chromatin and using 1% FGEL in blocking the beads. Enrichment was calculated relative to negative primer set. D) The effect of omitting SDS from the sonication buffer. Relative enrichment is calculated over non-transfected BirA control cells. Click here for file

Additional file 2 **GATA-1 ChIP of the GATA-2 gene locus.** A) Location of the ChIP primers in regulatory elements of the GATA-2 locus. B) GATA-1 binding using streptavidin M280 Dynabeads. C) The effect of blocking M280 Dynabeads with 1% FGEL. Enrichment was calculated relative to non-transfected BirA cells. Primer sequences are as published by Rodriguez et al. (ref. [8]). Click here for file
