# Supplementary figures and images for: Optimal use of tandem biotin and V5 tags in ChIP assays
Source: BMC Mol Biol. 2009 Feb 5;10:6. doi: 10.1186/1471-2199-10-6 (PMC2644297; doi:10.1186/1471-2199-10-6)

A.

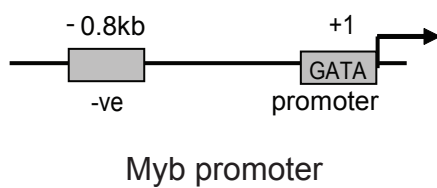

B.

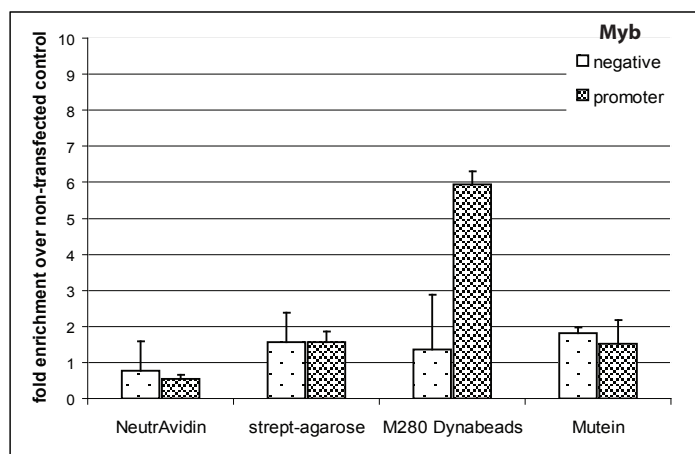

C.

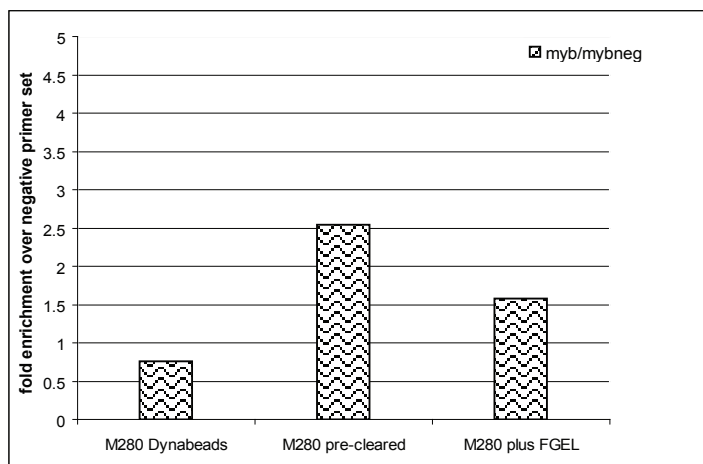

D.

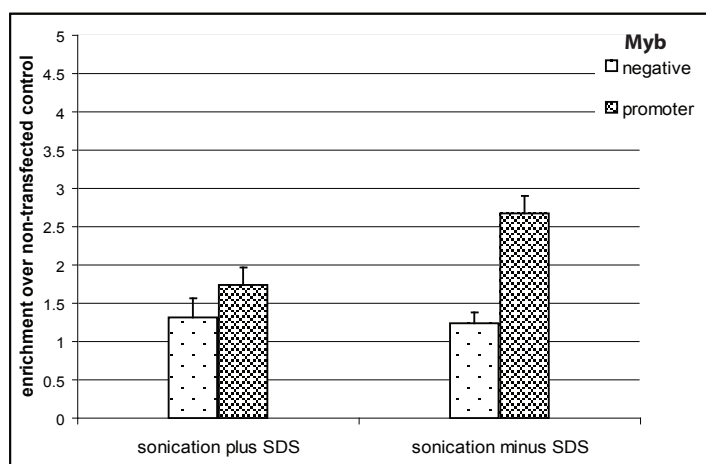

Supplement: Additional file 1 — GATA-1 ChIP of the myb promoter. A) Location of ChIP primers in the myb promoter. B) Comparison of different derivatives of immobilized streptavidin: NeutrAvidin, streptavidin agarose, streptavidin mutein and M280 Dynabeads. Relative enrichment is calculated over non-transfected BirA control cells. C) The effects of preclearing chromatin and using 1% FGEL in blocking the beads. Enrichment was calculated relative to negative primer set. D) The effect of omitting SDS from the sonication buffer. Relative enrichment is calculated over non-transfected BirA control cells. [file 1471-2199-10-6-S1.pdf]

A.

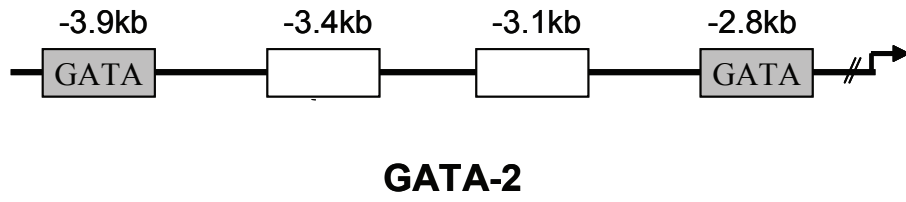

B.

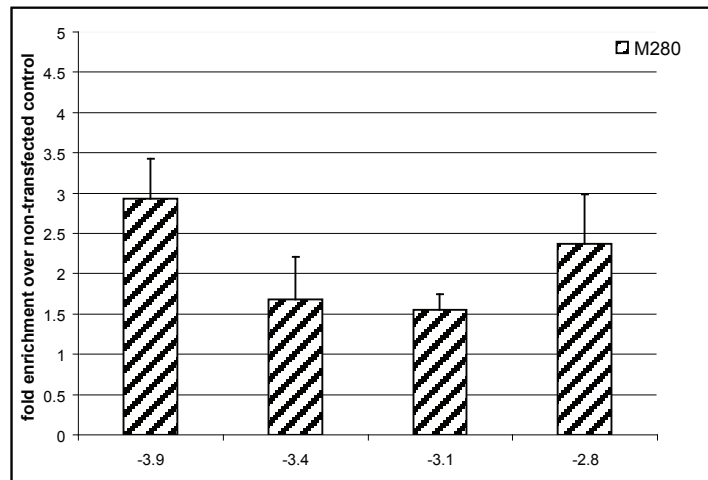

C.

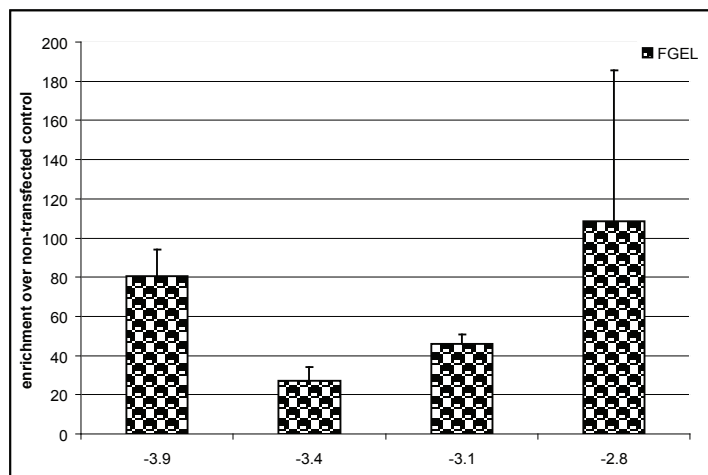

Supplement: Additional file 2 — GATA-1 ChIP of the GATA-2 gene locus. A) Location of the ChIP primers in regulatory elements of the GATA-2 locus. B) GATA-1 binding using streptavidin M280 Dynabeads. C) The effect of blocking M280 Dynabeads with 1% FGEL. Enrichment was calculated relative to non-transfected BirA cells. Primer sequences are as published by Rodriguez et al. (ref. [8]). [file 1471-2199-10-6-S2.pdf]
